# Association between systemic immune-inflammation index and low muscle mass in US adults: a cross-sectional study

**DOI:** 10.1186/s12889-023-16338-8

**Published:** 2023-07-24

**Authors:** Lin Shi, Liang Zhang, Dan Zhang, Zhuo Chen

**Affiliations:** 1 Department of Gastroenterology, Xuzhou Central Hospital, Xuzhou Clinical School of Xuzhou Medical University, Xuzhou, Jiangsu China; 2 Department of Gastrointestinal Surgery, Xuzhou Central Hospital, Xuzhou Clinical School of Xuzhou Medical College, Xuzhou, Jiangsu China; 3 Department of Nephrology, Affiliated Hospital of Xuzhou Medical University, 99 West Huai-hai Road, Xuzhou, 221002 Jiangsu China

**Keywords:** Systemic immune-inflammation index, Low muscle mass, Population-based study, NHANES, Cross-sectional study

## Abstract

**Background:**

Chronic inflammatory responses have been reported to be associated with low muscle mass and systemic immune-inflammation index(SII) is a novel indicator of inflammation. The purpose of our study was to clarify the relationship between SII and low muscle mass.

**Methods:**

This study was a cross-sectional study based on National Health and Nutrition Examination Survey (2011–2018). SII was calculated as the platelet count × neutrophil count/lymphocyte count. Appendicular skeletal muscle index was used to define low muscle mass. The individuals were divided into four groups by the quartile of SII (Q1-Q4). Multivariate weighted logistic regression analysis, smooth curve fitting and subgroup analysis were used to investigate the relationship between SII and sarcopenia. Subgroup analysis were based on demographic and clinical variables.

**Results:**

There were 10,367 individuals enrolled in our final analysis. The overall mean age was 39.39 years and 49.17% were males. The overall prevalence of low muscle mass in the study population was 8.77%. The smooth curve fitting analysis indicated a near-linear relationship between SII and low muscle mass. In multivariate weighted logistic regression analysis, the odds ratio (OR) of Q4 is 1.28 (95% CI, 1.16–1.40) for low muscle mass when compared to lowest quartile of the SII. In subgroup analysis, SII still increased the risk of low muscle mass independently.

**Conclusion:**

The increased SII levels were associated with an increased risk of low muscle mass in a large population. Our study increased the understanding between inflammation and low muscle mass. Anti-inflammation therapy may be important for low muscle mass.

## Introduction

Sarcopenia, a relatively common skeletal muscle disorder among the aging population, is featured by loss of the function and mass of skeletal muscle progressively, causing adverse outcomes, including increased risk of recurrent falls, fractures, physical disability, more frequent hospital services or hospitalization, decreased quality of life, or even death [1]. In addition to aging, sarcopenia is also associated with dementia, chronic obstructive pulmonary disease, liver cirrhosis cardiovascular disease [2–7].Low muscle mass is an important feature of sarcopenia and is also aging-related disorders [8]. In healthy middle-aged men and those with cardiovascular disease (CVD), there is compelling evidence demonstrating the autonomous and safeguarding impact of muscular strength on mortality from all causes and cancer [9]. The definite mechanisms and etiology of the pathophysiology of low muscle mass are not fully understood. Thus, there are no specific treatments or drugs available for low muscle mass [10, 11]. However, some studies have indicated that the inflammatory process may play an important role in the development of low muscle mass, which could affect the function and components of skeletal muscle [4, 12, 13]. A recent meta-analysis reported that higher inflammation biomarkers were observed in patients with lower muscle strength and muscle mass compared with the normal population.

Accumulating studies have demonstrated that inflammatory response was associated with many chronic diseases, tumorigenesis, and tumor development [12, 14, 15]. And growing studies have indicated that the systemic immune-inflammation index (SII) was an outstanding and steady indicator of the local immune response and systemic inflammatory response throughout the body [16]. The index was first proposed in 2014 by Hu.et al, however, it was soon widely and intensively used in clinical research [16]. SII is based on three easily accessible blood biomarkers, that are peripheral lymphocyte (Lym), neutrophil (Neu), and platelet (Plt) counts. The calculation formula is platelet count × neutrophil count/lymphocyte count [17]. Many current studies have found that SII is associated with a variety of diseases, such as recurrence and length of survival in hepatocellular carcinoma, colorectal cancer, lung adenocarcinoma, and prognosis in patients with aortic coarctation or other major cardiovascular and cerebrovascular diseases [18–21]. In addition, the SII index can also reflect the nutritional status of the dialysis population.

Because the inflammatory response is very closely related to the pathogenesis of muscle mass loss that it is well worth studying. And The relationship between the SII index and muscle mass is currently unknown. Therefore the purpose of our study was to clarify the relationship between SII and muscle mass among the participants of the US National Health and Nutrition Examination Survey (NHANES). We hypothesized that an elevated SII would be associated with a higher risk of low muscle mass.

## Materials and methods

### Data accession and study population

This study was based on the National Health and Nutrition Examination Survey (NHANES) 2011–2018 [22, 23]. The NHANES is conducted in the United States by the National Center for Health Statistics (NCHS) of the Centers for Disease Control and Prevention (CDC). All the data in NHANES were collected by unified trained professional personnel through household interview or an examination conducted in a mobile examination center [22, 24]. The survey consists of cross-sectional interviews, examination, and laboratory data collected from a complex multistage, stratified, clustered probability sample representative of the US population. The Institutional Review Board of the CDC has approved the survey protocol. All participants have provided informed consent [25–27].

In NHANES 2011–2018, subjects aged 20 years and over in the four consecutive study cycles were subjected to our study. In the four cycles, dual-energy X-ray absorptiometry (DXA) was used to measure body composition because of fast speed, low cost, and low radiation. However, individuals whose weight was > 136 kg or whose height was > 192 cm were excluded because the DXA scan had limitations on height ( ≦ 192.5 cm) and weight ( ≦ 136.4 kg); Moreover, pregnant women and subjects with a history of radiographic contrast material use or radioactive therapy in past 7 days were also excluded.

### Body composition, DXA and definition of low muscle mass

Body composition variables including height (cm), weight (kg), and waist circumference (cm) of the enrolled individuals were directly obtained from the 2011–2018 NHANES. Body mass index was calculated as described elsewhere.

DXA was performed on individuals aged 8–59 in NHANES 2011–2018 by Hologic Discovery model A densitometers (Hologic, Bedford, Massachusetts, USA). All non-fat and non-bone masses were regarded as skeletal muscle, while appendicular skeletal muscle mass (ASM) was defined as the sum of the lean soft tissues of the extremities. Appendicular skeletal muscle index (ASMI) was accepted to quantify muscle mass. The calculation formula is as follows: ASMI = total appendicular skeletal muscle mass (in kg)/BMI (kg/m2) [28]. Low muscle mass was defined by ASMI with the lowest sex-specific cut-off values: men were judged as low muscle mass if ASMI < 0.789, and women < 0.512 according to the National Institutes of Health [3].

### Systemic immune-inflammation index and other covariates

Demographic and laboratory data were obtained and merged from the NHANES database from 2011 to 2018. Demographic data included age, sex, and race (non-Hispanic white, non-Hispanic black, Hispanic, and others). We defined never smokers (smoked < 100 cigarettes during lifetime and does not smoke now), former smokers (smoked > 100 cigarettes during lifetime and does not smoke now), and current smokers (smoked at least 100 cigarettes in life and smoke now) according to self-reported smoking status. Drinking status was categorized as never (had < 12 drinks in a lifetime), former (had ≥ 12 drinks in 1 year and did not drink last year, or did not drink last year but drank ≥ 12 drinks in a lifetime), current light/moderate drinker (≤ 1 drink per day for women or ≤ 2 drinks per day for men on average over the past year), or current heavier drinker (> 1 drink per day for women or > 2 drinks per day for men on average over the past year). Current medical conditions were also included in our study based on self-reported forms or relevant laboratory or imaging findings: diabetes mellitus (yes/no), hypertension (yes/no), congestive heart failure(yes/no), stroke(yes/no), osteoarthritis(yes/no), osteoporosis(yes/no), cancer(yes/no), chronic kidney disease(CKD)(yes/no), and chronic obstructive pulmonary disease(COPD)(yes/no). The diagnosis of CKD was based on the estimated glomerular filtration rate [eGFR] < 60 ml/min per 1.73 m2 or urine albumin-to creatinine ratio (ACR) ≥ 30 mg/g [29, 30]. The diagnosis of osteoporosis was based on bone mineral density, which was evaluated by evaluated by dual-energy X-ray absorptiometry scans with Hologic QDR-4500 A fan-beam densitometers (Hologic, Inc., Bedford, Massachusetts). The threshold values of osteoporosis have been described elsewhere. They were 0.68, 0.59, 0.49, and 0.78 g/cm2 for the total femur, femur neck, trochanter, and intertrochanter, respectively [31]. The diagnosis of diabetes was based on any of the following criteria were met: (1) A self-reported diagnosis of diabetes. (2) Use of anti-diabetic drugs. (3) A Hemoglobin A1c (HbA1c) level ≥ 6.5% [25]. The diagnosis of hypertension was based on any of the following criteria were met: (1) A self-reported diagnosis of hypertension. (2) Use of anti-hypertensive drugs.3) Systolic blood pressure greater than 140mmHg or diastolic blood pressure higher than 90mmHg. Other medical conditions were based on self-reported medical history [32, 33]. Laboratory data included aspartate aminotransferase (AST), alanine aminotransferase (ALT), albumin, glycosylated hemoglobin (HbA1c), serum glucose, white blood cells (WBC), platelet (PLT), lymphocyte (Lym) and neutrophil (Neu). The systemic immune-inflammation index was defined as follows: SII = peripheral platelet counts × neutrophil counts/ lymphocyte counts. Detailed information on the specimen collection, processing, quality assurance, and monitoring are described elsewhere.

### Statistical methods

All analyses were conducted by R software(4.1.1). As the complex design of the NHANES survey, we followed the recommendations of NHANES, and a four-year cycle weights, stratification, and clustering were appropriately used in our study for each analysis. Categorical variables are expressed as numbers and weighted proportions. Continuous variables are presented as weighted means (Standard Error).

All enrolled participants were separated into 4 groups according to the quartile of SII. Baseline characteristics were compared between different groups by weighted linear regression for continuous variables and the design-adjusted chi-square test for categorical variables. Furthermore, to evaluate the independent relationship between SII and muscle mass status, multiple weighted regression analysis was used to adjust potential confounders and obtain odds ratios (ORs) and 95% confidence intervals (CIs). Model 1 had no covariate-adjusted; model 2 adjusted age, sex, and race; model 3 adjusted for age, sex, race, smoking, alcohol drinking, diabetes, hypertension, stroke, HbA1c, glucose, ALT, and AST. Moreover, to examine a potential nonlinear association between SII and muscle mass, restricted cubic splines (RCS) with adjustments for potential confounders were applied. The p-value for the nonlinearity of the smooth curve fitting was calculated. In our study, we conducted subgroup analysis based on the following considerations: (1) previous research indicating differences between subgroups such as sex, age ethnicity, smoking habit as muscle mass can be influenced by demographic factors and lifestyles [34, 35]. (2) the existence of distinct biological mechanisms, especially in some chronic diseases such as diabetes, hypertension, cancer etc [36, 37]. Chronic inflammation may play vital role in the development and progression of these disease, therefore we conducted subgroup analysis in individuals with and without these diseases. (3) In order to examine the robustness of our results. In our revised manuscript. Subgroups analysis on the associations of SII with muscle mass based on model 3 was conducted with stratified factors including sex (male/ female), age (< 60/≥ 60 years), race (non-Hispanic white, non-Hispanic black, Hispanic, and others), smoking (former/never/now), drinking(never, former, current light/moderate drinker or current heavy drinker, hypertension (yes/no), diabetes (yes/no), stroke (yes/ no), CKD (yes/no), cancer(yes/no), osteoarthritis(yes/no), and osteoporosis(yes/no). An interaction term was added to test the heterogeneity of associations between the subgroups as well. A p < 0.05 was considered statistically significant.

## Results

### Baseline characteristics of the study population

**Fig. 1:**
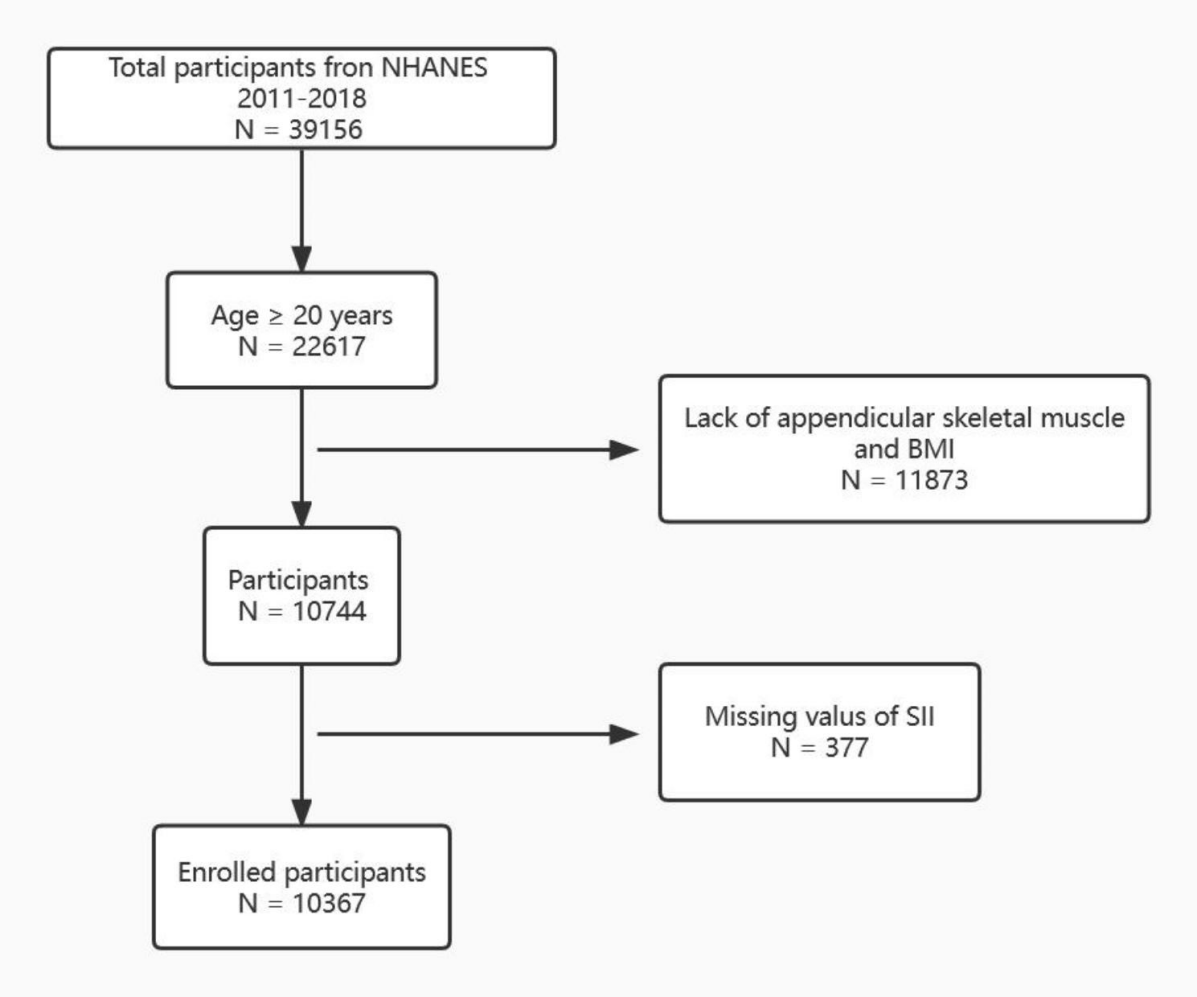
: The flow chart for our study

After screening the four cycles of NHANES, 10,367 individuals met our inclusion criteria and were enrolled in our final analysis. The detailed selection flow was presented in Fig. 1. The demographic data and laboratory indexes of the finally enrolled 10,367 participants were presented in Table 1. The study population was divided into 4 groups based on the quartile of SII. The first quartile group (Q1, n = 2592; SII ≦ 314.51562), the second quartile group (Q2, n = 2592; 314.41562 < SII ≦ 439.2), the third quartile group (Q3, n = 2591; 439.2 < SII ≦ 611.46265), and the fourth quartile group (Q4, n = 2592; SII > 611.46265). Of all the included individuals, the overall mean age was 39.39 years and 49.17% were males. The overall average of SII in our study was 508.61(5.07). The overall prevalence of low muscle mass in the study population was 8.77%. A progressive and significant increase in the prevalence of low muscle mass with SII levels was observed in Table 1 (p < 0.0001). Compared with the Q1 group, individuals in the Q4 group were more likely to be less male, Non-Hispanic white or black, and current smokers. Moreover, the frequency of hypertension, diabetes, CKD, osteoarthritis, and stroke increased gradually with increasing levels of SII (Table 1).

**Table 1:** Baseline characteristics of enrolled individuals from NHANES 2011–2018

		SII				
Variables	Overall	Q1	Q2	Q3	Q4	P-value
n	10,367	2592	2592	2591	2592	
Age, y	39.39(0.25)	39.02(0.42)	38.89(0.36)	39.64(0.34)	39.94(0.43)	0.002
Sex-Male, n (%)	5097(49.17)	1485(58.50)	1325(53.01)	1251(49.32)	1036(40.68)	< 0.0001
Race, n (%)						< 0.0001
Non-Hispanic White	3597(34.7)	640(52.71)	910(61.77)	1005(64.47)	1042(64.96)	
Non-Hispanic Black	2150(20.74)	851(19.48)	505(10.20)	385( 7.66)	409( 8.15)	
Mexican American	1563(15.08)	340(10.01)	404(10.81)	396(10.27)	423(11.28)	
Other	3057(29.49)	761(17.80)	773(17.22)	805(17.61)	718(15.62)	
Smoking, n (%)						0.01
Former	1727(16.67)	417(19.68)	459(20.84)	412(18.03)	439(19.17)	
Never	6318(60.97)	1628(60.36)	1613(60.12)	1588(59.20)	1489(56.44)	
Now	2318(22.37)	547(19.96)	517(19.04)	590(22.77)	664(24.38)	
Drinking, n (%)						0.02
Former	863(9.13)	206(7.81)	202(7.13)	214(8.55)	241(9.67)	
Never	1261(13.35)	332(10.67)	332(10.16)	293( 9.18)	304(10.13)	
Mild	3122(33.04)	845(36.57)	796(36.03)	759(32.76)	722(31.50)	
Moderate	1760(18.63)	395(18.38)	435(20.97)	469(21.90)	461(19.64)	
Heavy	2442(25.85)	590(26.56)	591(25.71)	616(27.61)	645(29.06)	
Diabetes, n (%)	1185(11.43)	254( 7.22)	283( 8.07)	298( 9.46)	350(10.67)	0.001
Hypertension, n (%)	2938(28.34)	687(24.62)	690(25.49)	756(27.49)	805(30.66)	0.01
Stroke, n (%)	154(1.49)	23(0.68)	44(1.28)	29(0.85)	58(1.78)	0.01
Congestive Heart Failure, n (%)	101(0.97)	20(0.43)	23(0.70)	21(0.57)	37(1.18)	0.02
COPD, n(%)	209(2.02)	44(1.90)	43(1.56)	47(2.22)	75(3.05)	0.09
CKD, n(%)	943(9.25)	192( 6.84)	205( 7.02)	227( 7.07)	319(10.44)	< 0.001
Cancer, n (%)	381(3.68)	75(4.29)	102(5.00)	95(4.98)	109(5.26)	0.73
osteoporosis	153(7.68)	30(4.03)	47(8.17)	42(8.08)	34(6.74)	0.46
osteoarthritis	530(5.31)	113(4.96)	128(6.92)	118(5.35)	171(7.99)	0.003
BMI, (kg/m^2^)	28.69(0.13)	27.54(0.18)	28.07(0.20)	29.23(0.20)	29.75(0.18)	< 0.0001
White blood cell, k/µL	7.32(0.04)	6.07(0.06)	6.80(0.05)	7.50(0.07)	8.73(0.07)	< 0.0001
Platelets, k/µL	242.38(1.03)	204.87( 1.27)	228.58( 1.18)	249.81( 1.36)	280.98( 1.58)	< 0.0001
Neutrophils, k/µL	4.31(0.03)	2.89(0.03)	3.77(0.03)	4.51(0.04)	5.89(0.06)	< 0.0001
Lymphocytes, k/µL	2.21(0.01)	2.45(0.03)	2.26(0.02)	2.18(0.02)	1.98(0.02)	< 0.0001
HbA1c (%)	5.52(0.01)	5.50(0.02)	5.50(0.03)	5.54(0.02)	5.54(0.02)	0.3
Glucose, mg/dL	104.48(0.61)	102.50(0.95)	103.76(1.04)	105.54(1.40)	106.36(1.01)	0.03
ALT (IU/L)	25.95(0.25)	26.63(0.63)	26.50(0.44)	26.25(0.53)	24.52(0.40)	0.01
AST (IU/L)	25.10(0.21)	26.22(0.51)	25.22(0.42)	24.69(0.34)	24.41(0.47)	0.16
Sarcopenia, n (%)	909(8.77)	164( 5.50)	202( 5.65)	240( 7.80)	303(10.09)	< 0.0001

### SII and muscle mass

To investigate the relationship between SII and muscle mass we developed three sample-weighted multivariate logistic models. The potential confounding factors which were adjusted in each model were presented in the method section. The odds ratio (OR) of Q4 is 1.28 (95% CI, 1.16–1.40), 1.28 (95% CI, 1.16–1.42), and 1.27 (95% CI, 1.17–1.43), respectively, in the model 1 (no adjustment), model 2 (adjusted for age, sex, and race), and model 3 (adjusted for age, sex, race, smoking, alcohol drinking, diabetes, hypertension, stroke, osteoarthritis, osteoporosis, CKD, COPD, HbA1c, glucose, ALT and AST). To ensure the accuracy of our results, we also performed a sensitivity analysis to compare the relations between different quartiles group. In model 3, the ORs were 0.98 (95%CI, 0.68–1.46) for Q2, 1.49 (95%CI, 1.01–2.18) for Q3, and 1.88 (95%CI: 1.37–2.58) for Q4 compared to Q1. The P value for the trend of the SII level was also calculated in each model by R software to examine whether a nonlinear relationship existed between muscle mass and SII, which is also shown in Table 2 (p for trend < 0.0001).

**Table 2:** Association between SII and low muscle mass among individuals in NHANES 2011–2018 – sample weighted multivariate logistic analysis

	OR (95%CI), P-value		
	Model 1	Model 2	Model 3
SII quartile as continuous	1.28(1.16,1.40), < 0.0001	1.28(1.16,1.42), < 0.0001	1.27(1.17,1.43), < 0.0001
SII quartile			
Q1	1	1	1
Q2	1.03(0.74,1.43), 0.86	1.00(0.71,1.42), 1	0.98(0.68,1.46), 0.97
Q3	1.46(1.05,2.02), 0.03	1.42(1.01,2.01), 0.04	1.49(1.01,2.18), 0.04
Q4	1.93(1.43,2.60), < 0.0001	1.92(1.38,2.65), < 0.0001	1.88(1.37,2.58), < 0.0001
P for trend	< 0.0001	< 0.0001	< 0.0001

Model 1: no adjustment

Model 2: adjusted for age, gender, and race

Model 3: adjusted for age, gender, race, smoking, alcohol drinking, diabetes, hypertension, stroke, osteoarthritis, osteoporosis, CKD, COPD, HbA1c, glucose, ALT, and AST.

**Fig. 2:**
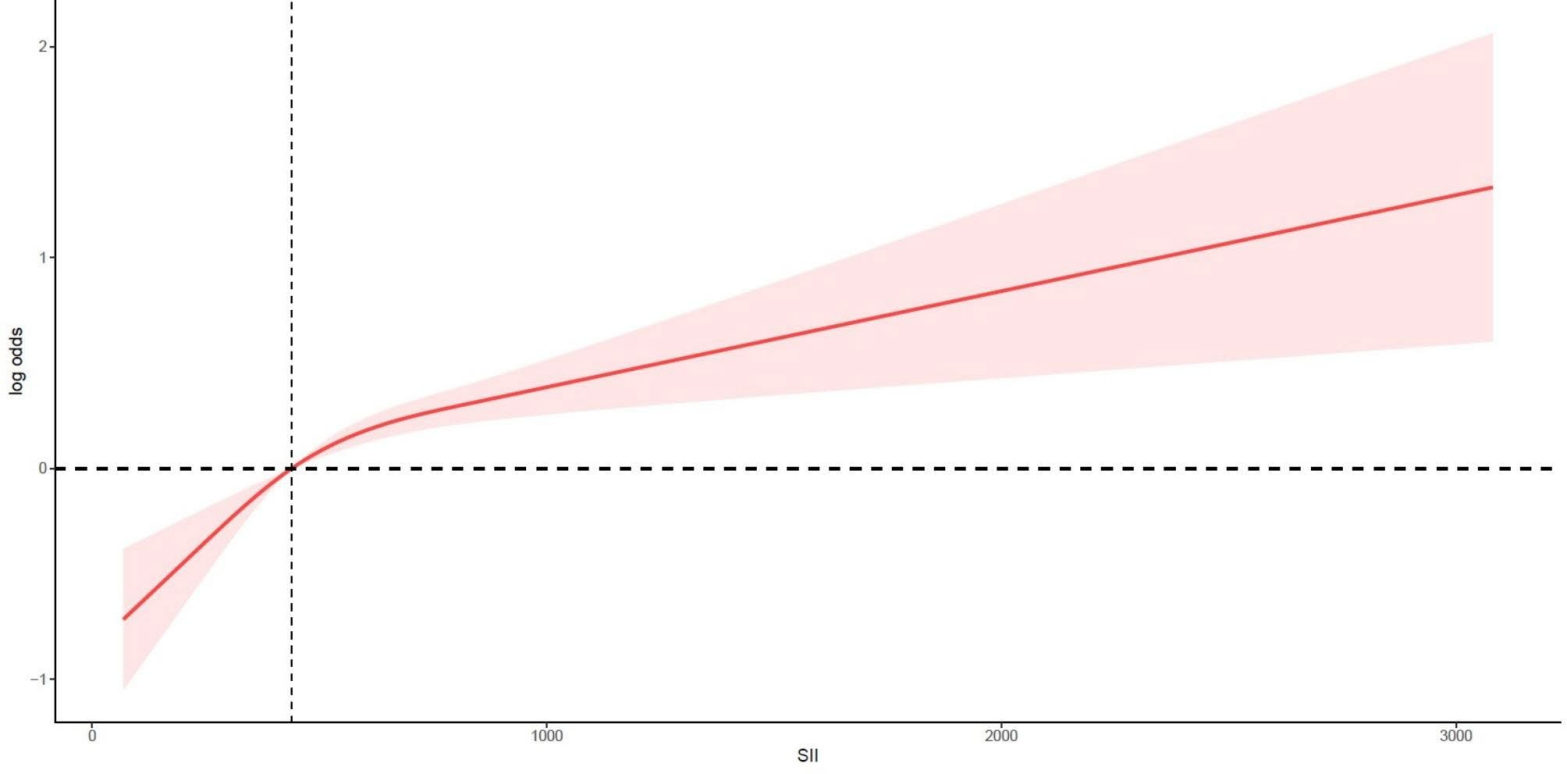
The smooth curve fitting between SII and low muscle mass

Moreover, the smooth curve fitting indicated a positive and near-linear relationship between SII and the risk of low muscle mass after adjusting all confounders (Fig. 2).

### Subgroup analysis

We conducted a subgroup analysis to examine the robustness of our results in each group. The detailed results of the subgroup were presented in Table 3 and the results were consistent in the subgroup. Several stronger associations between SII level and low muscle mass were observed in men, Non-Hispanic black, current smokers, and current alcohol users. And individuals with hypertension, diabetes, osteoarthritis, cancer and stroke were more likely to have a strong relation between SII levels and low muscle mass. No significant interactions between low muscle mass and SII level were observed in subgroup analysis, with all p for interaction exceeding 0.05.

**Table 3:** Subgroup analysis

Exposure	OR, 95%CI	p-value	p for interaction
Sex			0.69
Male	1.37(1.19, 1.56)	< 0.0001	
Female	1.21(1.06,1.36)	0.004	
Age			0.26
Age ≧ 60	1.33(1.17, 1.42)	< 0.001	
Age < 60	1.25(1.11,1.57)	< 0.01	
Race			0.66
Non-Hispanic White	1.21(1.03,1.49)	< 0.0001	
Non-Hispanic Black	1.79(1.28,2.45)	< 0.0001	
Mexican American	1.19(1.04,1.48)	0.03	
Other	1.41(1.22,1.69)	< 0.0001	
Smoking			0.99
Former	1.38(1.04,1.75)	0.02	
Never	1.21(1.08,1.48)	0.002	
Now	1.33(1.19,1.89)	0.003	
Drinking			0.69
Former	1.09(0.87,1.41)	0.41	
Never	1.13(0.77,1.45)	0.45	
Mild	1.5(1.20,1.84)	< 0.001	
Moderate	1.23(0.91,1.61)	0.21	
Heavy	1.35(1.01,1.99)	0.04	
Diabetes			0.44
Yes	1.31(1.08,1.58)	0.03	
No	1.25(1.12,1.39)	< 0.001	
Hypertension			0.6
Yes	1.27(1.09,1.49)	0.003	
No	1.26(1.13,1.39)	< 0.001	
Congestive Heart Failure			0.19
Yes	0.86(0.43, 1.70)	0.64	
No	1.28(1.16, 1.41)	< 0.0001	
CKD			0.31
Yes	1.41(1.12, 1.77)	0.004	
No	1.23(1.11, 1.36)	< 0.001	
COPD			0.29
Yes	1.66(1.05, 2.64)	0.03	
No	1.25(1.13, 1.38)	< 0.0001	
Cancer			0.51
Yes	1.45(1.04, 2.03)	0.03	
No	1.26(1.14, 1.40)	< 0.0001	
Osteoporosis			0.4
Yes	1.38( 0.77, 2.48)	0.26	
No	1.21(1.01, 1.46)	0.04	
Osteoarthritis			0.11
Yes	1.38(1.09, 1.74)	0.01	
No	1.26(1.11, 1.42)	< 0.001	
Stroke			0.29
Yes	1.49(0.79, 3.34)	0.34	
No	1.26(1.14,1.45)	< 0.0001	

## Discussion

In this present cross-sectional study, 10,367 individuals met our inclusion criteria and enrolled in our analysis. We found that subjects with higher SII levels showed an increased risk of low muscle mass. After adjusting all potential confounding factors, higher SII levels still independently increased the risk of low muscle mass. Our subgroup analysis and interaction test indicated that the relationship between SII levels and low muscle mass was robust in a different population setting. Although low muscle mass is more common in the elderly, we still found a proportion of middle-aged people with low muscle mass in our study, and SII was still independently associated with low muscle mass in the subgroup analysis of different ages.

The relationship between inflammation and muscle metabolism had been discussed in many studies and the mechanisms of the process have not been fully elucidated. Previous studies have reported that chronic inflammation was associated with decreased skeletal muscle mass and loss of muscle function [4]. Many studies have reported that SII is associated with various studies. A population-based cross-section study, using the NHANES database, found that higher SII level was associated with increased urinary albuminuria excretion independently with enrolling 36,463 participants in the study [17]. Similarly, in a Chinese cohort study that included 218 patients with severe pancreatitis, the SII level was found to be a good and timely predictor of acute kidney injury [38]. Moreover, it is reported in a newly published retrospective study that the SII index, calculated in hospital admission, independently predicted the development of vasospasm [39]. The optimal SII cutoff point in that study was 1.924 103/µL and SII could have a better predictive performance incorporated with age, aneurysm location, diabetes mellitus, and hyperlipidemia [39]. Another retrospective study in China with 496 patients reported that preoperative SII is significantly associated with postoperative survival in patients with acute type A aortic dissection undergoing surgical repair [18]. In the field of oncology research, SII has been reported as a good indicator of postoperative acute kidney injury in hepatocellular carcinoma patients as well [40]. In patients with metastatic or unresectable colon cancer, the higher the SII index, the shorter the survival period. And a high SII index may predict that liver metastases from colon cancer can occur at an early stage [20]. A meta-analysis included 3565 patients with esophageal cancer patients receiving operation in nine studies indicated that higher SII was associated with a shorter length of survival [21]. However, some studies also revealed the limitations of the SII index. A study involving 378 patients who underwent kidney transplantation indicated that the SII index has a limited role in predicting the prognosis of kidney transplant patients. The performance of the SII index was improved until adding other clinical indicators. A new meta-analysis showed that diabetes and diabetes complications were associated with a higher risk of sarcopenia compared to the normal population. In our subgroup analysis, individuals with diabetes had a stronger association with sarcopenia compared with those without diabetes. Our study was consistent with those studies that higher SII levels were associated with an increased risk of sarcopenia.

Although the SII index is a good indicator of inflammation in the body, there are other indicators of inflammation in clinical practice for assessing the association between inflammation and muscle metabolism: C-reactive protein (CRP), interleukin 6 (IL-6) and tumor necrosis factor (TNFα). CRP is usually elevated in acute and chronic inflammatory responses, reflecting the inflammatory state of an individual. Moreover, an increased serum concentration of CRP was associated with a high incidence of many diseases such as chronic kidney failure, type 2 diabetes, cardiovascular disease, sarcopenia, loss of muscle mass, and strength and poor physical performance [41]. In a meta-analysis including 1370 individuals, the authors reported that patients with sarcopenia had higher concentration of CRP compared with controls [15, 26, 41]. Camilla et al. also reported that a stronger relationship between CRP and muscle mass than IL-6 was observed in the population living in the community [4]. Although CRP could reflect the inflammatory status of the whole body, when a patient is combined with other chronic inflammatory diseases, such as rheumatoid arthritis, some cytokines such as interleukin 6 can act directly on muscle tissue, causing a decrease in muscle strength and mass [37]. IL-6 has long been considered as a promoter of muscle anabolism or catabolism in vivo environment [42]. Moreover, chronic inflammation can activate the IL-6 pathway, resulting in persistently elevated IL-6 concentration. Although elevated concentrations of interleukin-6 in a limited time could contribute to improving the glucose tolerance of muscle fiber as well as insulin sensitivity [43, 44], sustained high concentrations of interleukin-6 could cause muscle cell atrophy by disturbing muscle anabolism and energy homeostasis [42]. In addition, a meta-analysis examining the relationship between IL-6 and grip strength also found that elevated interleukin was associated with decreased grip strength [45]. TNFα is a classical pro-inflammatory cytokine that can act directly on skeletal muscle cells through two TNF receptors, TNFR1 and TNFR2, which are constitutively expressed in skeletal muscle fibers [36]. The negative association between TNFα and muscle mass was also observed in Camilla et al’s report. Moreover, inflammation and pyroptosis also play important roles in the development of sarcopenia [46]. Activation of the NLRP3 inflammasome due to inflammation can cause pyroptosis, which leads to cell death [47]. NLRP3 activates caspase-1, which then acts on gasdermin D (GSDMD) [48]. This causes membrane pores to form, leading to ion flux and the release of ATP, HMGB1, and interleukin (IL)-1β into the cell. This process ultimately results in cell death [48]. The NLRP3 inflammasome and pyroptosis can cause muscle dysfunction by reducing the glycolytic potential and myofiber size [46, 47]. Given this perspective, there is a need for new therapeutic approaches to reduce inflammation and pyroptosis in muscle metabolism.

One thing we still need to note is that the prevalence of reduced muscle mass is higher in the male population and inflammatory markers such as CRP, IL-6, and TNFα are more closely associated with reduced skeletal muscle mass than in women of the same age [4, 41]. In our study, we also observed males have a stronger association with reduced muscle mass compared with females (Males: OR = 1.38; 95%CI, 1.20–1.60; p = < 0.0001; Females: OR = 1.20; 95%CI, 1.06–1.37, p = 0.005).

On balance, the SII index can be a good and accurate predictor and biomarker of various diseases, while the low cost, non-invasive and easy availability of the SII index make it a broad application prospect.

Our study has several strengths and limitations. To our knowledge, our study is the first study to assess the association between SII and low muscle mass in a general population. Moreover, we had a large sample size with representative sample selection, which can help adjust for more confounding factors to produce more reliable results. But as an observational and cross-sectional study design, our study also has several limitations. First, as a cross-sectional analysis, a definite causal relationship is difficult to obtain and the self-reported medical status may introduce recall bias, therefore a prospective study is needed. Second, due to the lack of muscle strength data in NAHNES, muscle strength was not included in our study. Future research would use longitudinal study designs to include such variables and better study the mechanisms between inflammation and muscle metabolism. In addition, although we have adjusted for several confounding factors, we still cannot completely rule out other confounding factors in the analysis and subgroup analysis.

## Conclusion

In conclusion, in this cross-sectional study, we demonstrated that increased SII levels were associated with increased risk of low muscle mass in a nationwide population. Further prospective studies are needed to validate this finding.

Legends:

## Data Availability

Publicly available datasets were analyzed in this study. This data can be found here: https://www.cdc.gov/nchs/nhanes.
